# The landscape of implantation and placentation: deciphering the function of dynamic RNA methylation at the maternal-fetal interface

**DOI:** 10.3389/fendo.2023.1205408

**Published:** 2023-08-30

**Authors:** Shengyu Wu, Han Xie, Yao Su, Xinrui Jia, Yabing Mi, Yuanhui Jia, Hao Ying

**Affiliations:** ^1^ Department of Clinical Medicine, Tongji University School of Medicine, Shanghai, China; ^2^ Department of Obstetrics, Shanghai Key Laboratory of Maternal Fetal Medicine, Shanghai Institute of Maternal-Fetal Medicine and Gynecologic Oncology, Shanghai First Maternity and Infant Hospital, School of Medicine, Tongji University, Shanghai, China; ^3^ Clinical and Translational Research Center, Shanghai Key Laboratory of Maternal Fetal Medicine, Shanghai First Maternity and Infant Hospital, School of Medicine, Tongji University, Shanghai, China

**Keywords:** implantation, placentation, epigenetic modification, post-transcriptional regulation, RNA methylation, maternal-fetal interface

## Abstract

The maternal-fetal interface is defined as the interface between maternal tissue and sections of the fetus in close contact. RNA methylation modifications are the most frequent kind of RNA alterations. It is effective throughout both normal and pathological implantation and placentation during pregnancy. By influencing early embryo development, embryo implantation, endometrium receptivity, immune microenvironment, as well as some implantation and placentation-related disorders like miscarriage and preeclampsia, it is essential for the establishment of the maternal-fetal interface. Our review focuses on the role of dynamic RNA methylation at the maternal-fetal interface, which has received little attention thus far. It has given the mechanistic underpinnings for both normal and abnormal implantation and placentation and could eventually provide an entirely novel approach to treating related complications.

## 1 Introduction

The maternal-fetal interface is the interface between maternal tissue and fetal components in direct contact. It is primarily made up of the placenta and maternal decidua (1). At the cellular level, these structures are predominantly composed of trophoblast, decidual, and immune cells (2). Additionally, the normal early embryonic growth is necessary for the development of the maternal-fetal interface. Poor maternal-fetal interface development predisposes to a variety of unfavorable pregnancy outcomes, like miscarriage and preeclampsia (3, 4).

RNAs play a crucial role in controlling biological functions. They have emerged as key regulators of RNA function and metabolism (5). In eukaryotes, RNA methylation modifications are the most common type of RNA alteration in eukaryotes, accounting for 60% of all RNA modifications (6). N6-methyladenosine (m6A), N1-methyladenosine (m1A), 5-methylcytidine (m5C), and others are prevalent types of RNA methylation. The most common type of RNA methylation on messenger RNAs (mRNAs) in complex organisms is m6A RNA methylation (7). Normal and abnormal implantation and placentation are both affected by RNA methylation. By influencing the early stages of embryo development, trophoblast invasion, endometrial receptivity, and immunological microenvironment, it is essential for the establishment of the maternal-fetal interface (8). Furthermore, preeclampsia and other disorders associated with implantation and placentation are closely correlated with RNA methylation (9).

**Figure 1:**
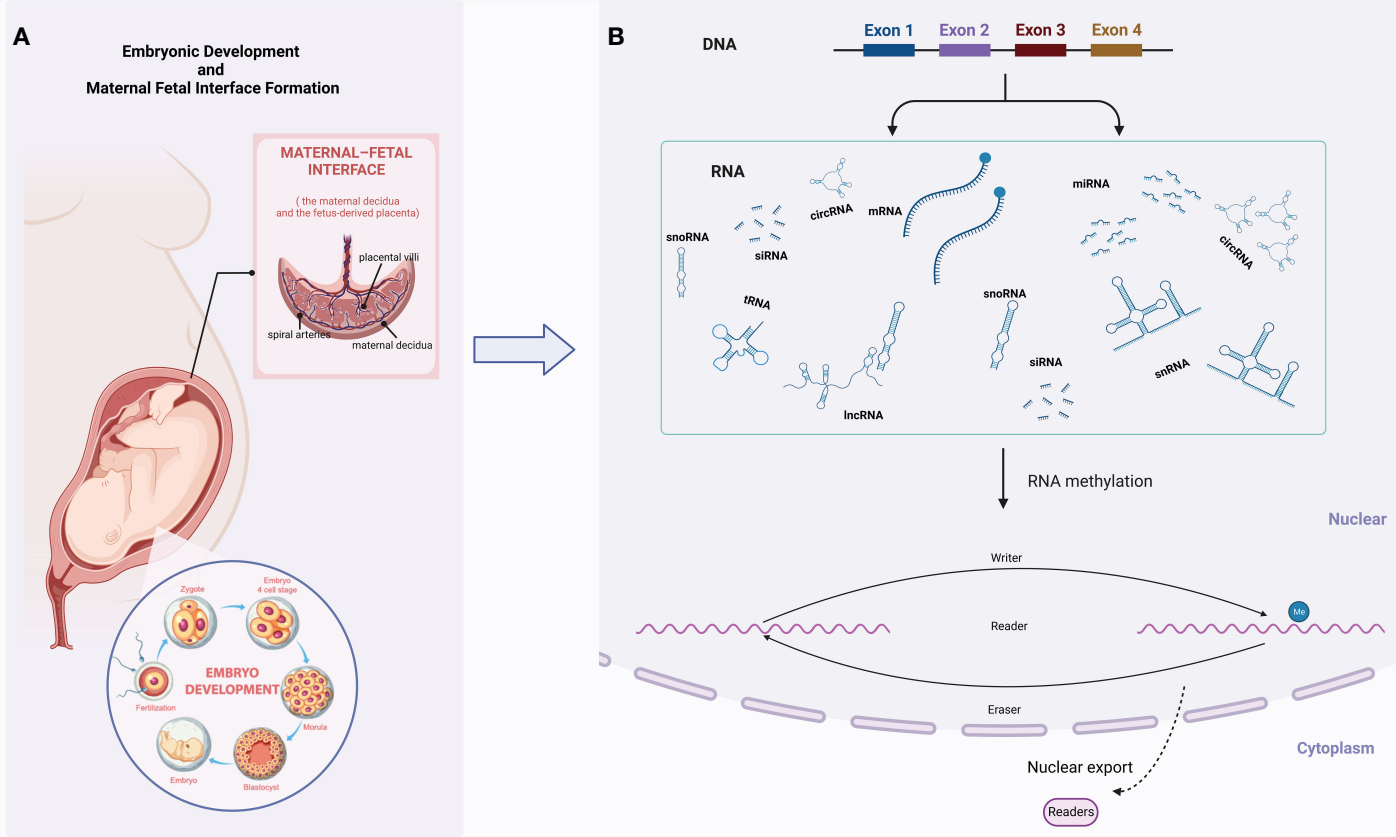
Graphical abstract. **(A)** Embryonic development and maternal-fetal interface formation; **(B)** RNA methylation.

Despite significant breakthroughs in the study of pregnancy, it is still exceedingly challenging to comprehend how RNA methylation works in human implantation. Our review covers the most recent RNA methylation studies and discusses the several forms of RNA methylation in humans, as well as the role of regulatory factors. Furthermore, we discuss the significance of dynamic RNA methylation in implantation and placental development. In addition, our review highlights the previously unknown involvement of RNA methylation in the formation of the maternal-fetal interface. In brief, this paper aims to review recent findings on developmental changes at the maternal-fetal interface associated with RNA methylation and its implications for implantation and placenta-associated diseases. This review is crucial because it provides a comprehensive overview of the current state of research on RNA methylation at the maternal-fetal interface, points to future research directions, and offers the prospect of a new strategy for the future treatment of related disorders (**Figure 1**).

## 2 Common types of RNA methylation modification in homo species

In both prokaryotes and eukaryotes, over 100 different types of post-transcriptional chemical modifications of RNA have been discovered in messenger RNA (mRNA), ribosomal RNA (rRNA), transfer RNA (tRNA), and small RNA (10, 11). RNA methylation is one of the most major epigenetic modifications in post-transcriptional RNA modification (12–14). It is estimated that methylation is responsible for more than 60% of RNA alterations (15). They all play critical roles in metabolism, structural stability, and RNA synthesis (**Table 1**) (85, 86).

**Table 1:** Regulators in coding and non-coding RNA modification.

RNA		Regulators	Modification		
			m6A	m5C	m1A
Coding RNA	mRNA	Writer	METTL3 (1997) (16) METTL14 (2014) (17, 18) WTAP (2014) (17, 18) KIAA1429(2014) (19) RBM15(2016) (20) METTL16(2017) (21) ZC3H13(2018) (22) VIRMA (2018) (22) CAPAM (2019) (23) CBLL1(2021) (24)	NSUN2(2012) (25) NSUN6 (2020) (26)	TRMT10C (2017) (27)
		Eraser	FTO (2011) (28) ALKBH5 (2013) (29)	TET2 (2016) (30) TET3 (2014) (31)	ALKBH3 (2022) (32)
		Reader	HNRNPA1 (2007) (33) HNRNPA2B1 (2015) (34) HNRNPC (2015) (35) YTHDF1 (2015) (36) YTHDF2 (2016) (37) YTHDC1 (2016) (38) YTHDF3 (2017) (39) YTHDC2 (2017) (40) FMR1 (2017) (41) LRPPRC (2017) (42) RBMX (2017) (43) IGF2BP1/2/3 (2018) (44) ELAVL1 (2019) (45)	ALYREF (2006) (46) YBX1 (2014) (47)	/
Non-coding RNA	tRNA	Writer	METTL16 (2017) (48) METTL5:TRMT112 (2021) (49)	NSUN2 (2012) (50) NSUN6 (2020) (26)	TRMT10C (2017) (27)
		Eraser	/	TET2 (2021) (51) TET3 (2014) (31)	ALKBH1 (2016) (52) FTO (2018) (53) ALKBH3 (2019) (54)
		Reader	/	YBX1 (2018) (55)	/
	rRNA	Writer	METTL16 (2017) (48) METTL5 (2019) (56) ZCCHC4 (2020) (57)	NOP2 (2010) (58)	TRMT10C (2017) (27)
		Eraser	/	TET3 (2014) (31)	/
		Reader	/	YTHDF2 (2020) (59)	/
	microRNA	Writer	METTL3 (2015) (60) METTL14 (2017) (56) METTL16 (2017) (48) METTL4 (2022) (61)	/	/
		Eraser	ALKBH5 (2021) (62) FTO (2021) (63)	TET3 (2014) (31)	/
		Reader	HNRNPA1 (2007) (64) HNRNPA2B1 (2015) (34) ELAVL1 (2018) (65) IGF2BP1 (2018) (66) IGF2BP2 (2018) (66) IGF2BP3 (2018) (66) FMR1 (2021) (67) YTHDC1 (2021) (68) HNRNPC (2022) (69)	/	/
	lncRNA	Writer	METTL3 (2016) (20) RBM15 (2016) (20) METTL16 (2017) (48) METTL14 (2020) (70) VIRMA (2021) (71) WTAP (2022) (72)	NSUN2 (2020) (73)	TRMT10C (2017) (27)
		Eraser	ALKBH5 (2019) (74) FTO (2022) (75)	TET3 (2014) (31)	/
		Reader	IGF2BP1 (2013) (76) HNRNPC (2015) (35) YTHDC1 (2016) (20) RBMX (2017) (43) ELAVL1 (2019) (77, 78) HNRNPA2B1 (2020) (79)	/	/
	circRNA	Writer	METTL3 (2017) (80) METTL16 (2017) (48) METTL14 (2022) (81)	/	/
		Eraser	/	TET3 (2014) (31)	/
		Reader	FMR1 (2006) (82) YTHDF3 (2017) (80) HNRNPA2B1 (2022) (83) YTHDC1 (2020) (84)	/	/

/, Not available.

### 2.1 m6A modification in RNA

As early as 1977, Karen Beemon et al. found residues of m6A in Rous sarcoma virus RNA (87). Since two groundbreaking papers in 2012 demonstrated the distribution of m6A residues in the mammalian species, there has been an increase in interest in RNA-methylation-based research (88, 89). Previous studies reveal that m6A modification occurs preferentially at the m6A consensus motif RRACH, but not all RRACH sequences will have m6A modification (90). The dynamic network of m6A is regulated by methyltransferase complex (“writer”) and demethylase (“eraser”), with “writer” adding m6A modification and “eraser” exerting demethylation activity (91). Moreover, the downstream functions of m6A are mediated by RNA-binding proteins (“reader”) that recognize m6A and regulate RNA processing (35, 92).

METTL3 is the first methyltransferase discovered and it is one of the most fundamentally important “writers” (16). METTL14 and METTL3 share regions of sequence similarity (>35% nucleotide sequence identities) (93). They can form a heterodimeric core complex, with METTL3 acting primarily as a catalytic core and METTL14 acting as an RNA binding platform (94–96). The solution structure of the zinc finger structural domain (ZFD) of METTL3 fulfills the methyltransferase activity of the complex (97). The METTL3- METTL14 dimer mediates the deposition of m6A on nuclear RNA in mammalian cells (17). WTAP is another regulatory subunit of m6A methyltransferase in RNA (18). WTAP itself has no catalytic activity for m6A modification because it lacks a conserved catalytic methylation domain. However, it acts as a bridging protein for the interaction with METTL3 and METTL14, thus significantly affecting RNA m6A loading (17). The modification can be reversed by FTO and ALKBH5 since they are chief erasers that mediate the m6A demethylation process (28, 29). In addition, the m6A “readers” mainly belong to the YT521-B homology (YTH) family and contain the YTH structural domain (98). IGF2BPs are a recently identified class of m6A readers that preferentially recognize m6A-modified mRNAs and help to maintain the stability of thousands of possible mRNA targets, including MYC (44).

A growing body of evidence suggests that m6A is involved in RNA structural stability, translation, and degradation at the mRNA level (99–103). It can modulate protein binding by inducing RNA structural changes that alter the accessibility of the protein binding site (43). Additionally, m6A modification alteration serves several functions in linking pre-mRNA maturation to mRNA fate. Recent studies have shown that m6A in mRNA appears to be involved in the regulation of selective splicing, translation efficiency, and stability. m6A also controls the lifetime and degradation of mRNA. There is sufficient evidence that the YTH structural domain family promotes mRNA degradation. YTHDF1 interacts with AGO2 via the YTH structural domain and then undergoes liquid-liquid phase separation (LLPS) leading to mRNA degradation by P-body formation (104).

Similar to mRNA, m6A-related modifications have also been found to be involved in non-coding RNA synthesis and metabolism (60, 105). METTL3 can methylate chromosome-associated regulatory RNAs (carRNAs), such as promoter-associated RNAs (paRNAs), enhancer RNAs (eRNAs), and RNAs produced from transposable elements (repeat RNAs). These carRNAs are destabilized by m6A methylation, which affects adjacent chromatin states and downstream transcription (106).

In addition, the RNA writers, erasers, or readers mentioned above are thought to be involved in DNA methylation processes. FTO and ALKBH5, two m6A erasers, may also be related to DNA methylation. Evidence suggests that ALKBH5 demethylates DNA’s 3-methylcytosine and has limited DNA repair activity (107). FTO, however, has a negligible impact on the demethylation of 5-methyl-2’-deoxycytidine (5mdC) in DNA (108). However, this might lead to intriguing new directions for an interaction between DNA methylation and RNA methylation regulators. Recent studies have also shown that RNA m6A methylation can reverse chromatin remodeling and histone alterations. Shuang Deng et al. discovered that RNA m6A modifier reader FXR1 can recruit the DNA 5-methylcytosine dioxygenase TET1 to genomic areas and remove DNA methylation (109, 110).

### 2.2 m5C modification in coding RNA and non-coding RNA

Aside from m6A, m5C is another common modification found in RNAs. During m5C modification, “writers”, “erasers”, and “readers” may act through similar mechanisms compared to m6A (111). Interestingly, recent studies have revealed that the m6A and m5C modifications of the same RNA may interact and synergistically affect protein expression patterns (112, 113). m5C can be installed by any of the seven proteins of the Nol1/Nop2/SUN domain (NSUN) family as well as DNMT2 (114).

As an important epigenetic modifier in RNA, m5C plays a central role in governing transcript abundance. NOP2/NSUN1, a m5C RNA methyltransferase, inhibits transcription and promotes viral latency by competing with TAT for TAR binding and methylation (115). m5C RNA methyltransferase can bind to and stabilize mRNA. As a typical tRNA methyltransferase, methylation of p16 3’UTR by NSUN2 may stabilize p16 mRNA (116). m5c modification has also been shown to regulate the nucleo-plasmic shuttling mechanism of RNA. NSUN2 can regulate the nucleo-plasmic shuttle and RNA binding of ALYREF. After NSUN2 knockdown, nuclear retention of ALYREF is enhanced and, subsequently, cytoplasmic localization is reduced (117). In mammals, ALYREF recognizes and exports YBX2 and SMO mRNAs with m5C modifications from the nucleus to the cytoplasm (118). These findings strongly suggest that m5C is specifically recognized by ALYREF and thus promotes selective mRNA export. In addition, cytosine methylation level and the function of RNA translation are interdependent (119). m5C methylation increases translation levels in NSUN6-targeted mRNAs. Further evidence from ribosome analysis shows that NSUN6-specific methylation is connected to translation termination (120).

Additionally, these RNA m5C methylation-related writers, readers, and erasers might also be involved in DNA methylation. For example, DNA methylation is carried out by members of the DNA methyltransferase (DNMT) family (121). However, mammalian DNMT2 is also a tRNA methyltransferase with a conserved tRNA recognition mechanism (122). DNMT2-mediated m5C contributes to the secondary structure and biological properties of small non-coding RNAs (sncRNAs) and promotes the ability of sperm RNA to induce metabolic changes in offspring (123).

m5C alterations are also involved in the maturation and structural changes of non-coding RNAs. Based on miCLIP-Seq data, Han Liao et al. discovered that NOP2/NSUN1 catalyzes the deposition of m5C on 28S rRNA and regulates the processing of pre-rRNA (124). The NSUN4-mTERF4 complex is involved in the maturation of 16S rRNA (125), while NSUN5 is associated with overall protein synthesis and normal growth (126).

m5C modification in RNA is considered to be reversible. Demethylation is carried out by ten-eleven translocation (TET) enzymes. Although the catalytic activity of TET proteins was thought to be limited to DNA for several years, current research has begun to uncover their potential significance in altering RNA bases. TET2 has been shown to promote the conversion of 5-methylcytosine on tRNA to hm5C as well as to regulate the processing and stabilization of different classes of tRNA fragments (51). This may be related to intracellularly mediated m5C oxidation by TET2 (127, 128).

Furthermore, readers are responsible for reading the information on RNA m5C modification. ALYREF was characterized as a nucleoplasmic mRNA m5C reader (117). Further studies indicated that CDKN1A is an ALYREF target and a reader of m5C-modified mRNA (129). In humans, FMRP has been identified as a novel “reader” of m5C modification, promoting mRNA-dependent repair and cell survival in cancer (130). Moreover, YTHDF2 can also be considered as a type of m5C reader. It can bind directly to m5C in rRNA based on Trp432, a conserved residue located in the hydrophobic pocket of YTHDF2 (59).

Overall, the functional roles of m5C modification involve pre-RNA processing and splicing, mRNA stability, and other processes (131–135). However, the detailed mechanism is not clear. In addition, current research on m5C demethylases is restricted, and its role in RNA has to be investigated further.

### 2.3 m1A modification in RNA

As a novel form of RNA modification, m1A has received considerable attention in recent years. m1A is mainly present in the T-loop structure of tRNA and is introduced by the TRMT6/TRMT61A complex. It appears less commonly in mRNA. Modi Safra et al. discovered a m1A site catalyzed by TRMT10C in mitochondrial ND5 mRNA with high tissue-specific and tight developmental control of methylation levels (27).

Erasers act as m1A demethylases, removing the methyl group from m1A and making it functionally reversible. ALKBH1 and ALKBH3 are two common demethylases (52, 54).

Using a photocrosslinking approach, Seo KW et al. discovered that YTHDF1/2 is the reader of m1A modification on RNA and that the binding of YTHDF2 to m1A promotes the degradation of m1A RNA (136).

Nevertheless, little is known about m1A development pathways, and their function in RNA remains to be elucidated.

Notably, over the last few years, more than 100 alterations inside RNAs have been discovered, including m1G, m6G, and others. Further inquiry is required to determine their role in molecular function.

## 3 RNA methylation in the formation of the maternal-fetal interface

During the establishment of pregnancy, early embryonic development and implantation are important components (137). Normal preimplantation embryo development is a crucial step for a successful pregnancy. The human blastocyst begins to implant in the uterus seven days after fertilization by adhering to the receptive endometrium via its trophectoderm (138).

During the first trimester of human pregnancy, an interface is formed between the maternal decidua and the placenta of the fetus, which is also known as the maternal-fetal interface (139). It is a unique microenvironment composed of the maternal decidua and fetal trophoblast. Immune crosstalk at the maternal-fetal interface is also required for a successful pregnancy (1). Trophoblast, decidual stromal cells, and immune cells are important in metaphase vascularization, maternal immune tolerance, and maternal metabolic changes that facilitate fetal nutrient delivery (4).

**Figure 2:**
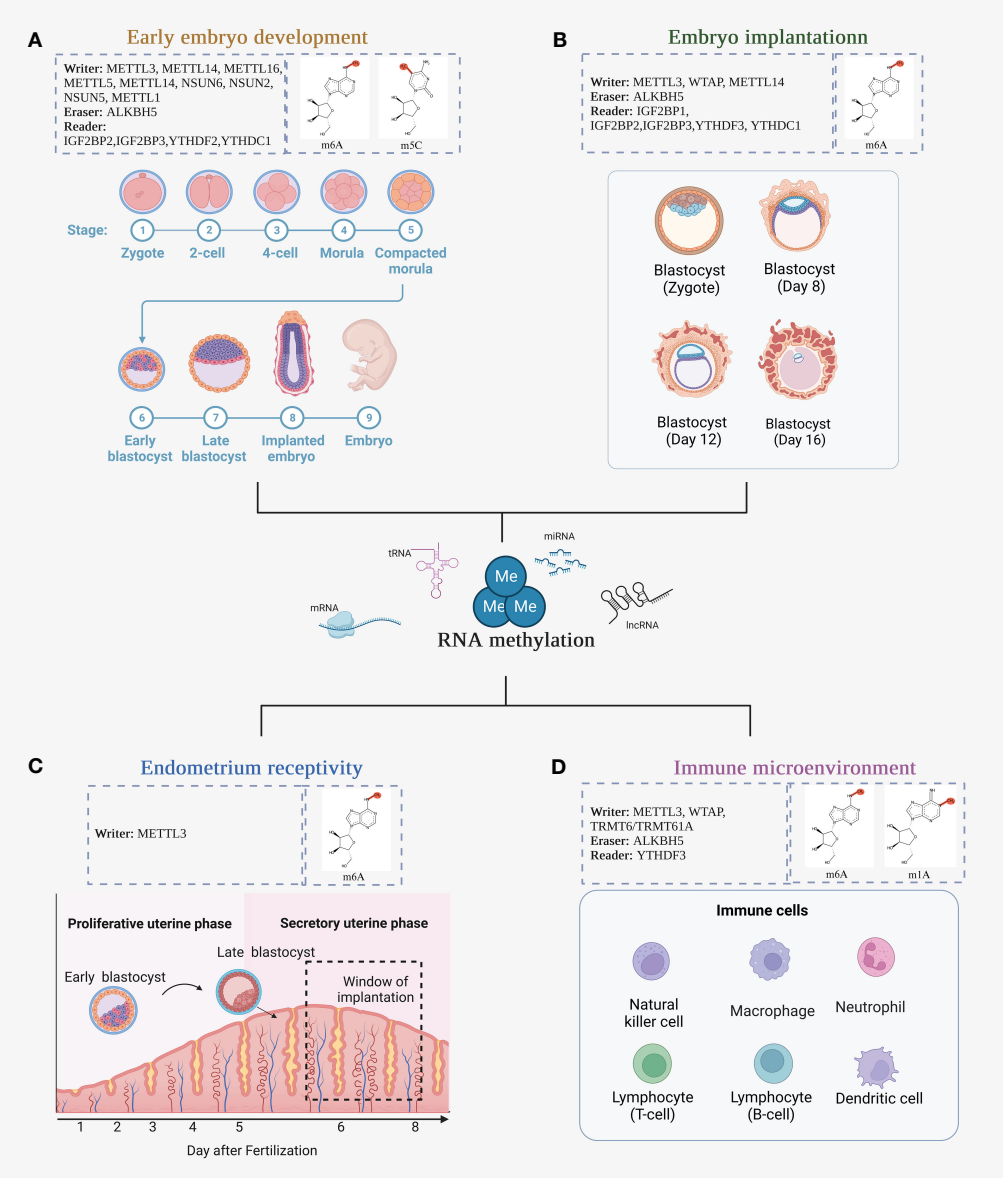
The function of RNA methylation in the formation of the maternal-fetal interface. **(A)** Early embryo development. Early embryonic development undergoes a transition from the zygote, 2-cell stage, 4-cell stage, morula, compacted morula, early blastocyst, late blastocyst, implanted embryo, and embryo; **(B)** The changes in blastocyst during embryo implantation; **(C)** The establishment of endometrium receptivity; **(D)** Different immune cells involved in the immune microenvironment.

RNA methylation may have a major role in the development of the maternal-fetal interaction. In this section, we summarized the function of RNA methylation in this section, focusing on four aspects in particular: early embryo development, embryo implantation, endometrial receptivity, and immunological microenvironment (**Figure 2**).

### 3.1 RNA methylation in the early embryo development

Semen RNA epigenetic modifications have potential effects on early embryogenesis and paternal epigenetic inheritance (140). METTL3-mediated m6A regulation is critical for male fertility and spermatogonia differentiation during spermatogenesis (141). It also modulates protein synthesis in spermatogonial stem cells and spermatids. Dual deletion of METTL3 and METTL14 in advanced germ cells can result in poor spermatogenesis due to altered translational efficiency of m6A-containing transcripts (142). Furthermore, ALKBH5-mediated m6A erasure is required for appropriate splicing and production of longer 3’-UTR mRNAs, and reversible m6A modification is a key mechanism for post-transcriptional control of mRNA fate in late meiotic and haploid germ cells (143). These studies hint to the impact of RNA alteration on sperm development, which is responsible for the normal development of the embryo after fertilization.

Maternal RNA degradation, comprising maternal (M) and zygotic (Z) decay pathways, is also essential for oocyte maturation and embryogenesis. YAP and TUT4/7 may regulate maternal mRNA Z-decay during the human maternal-to-zygote transition (MZT) and play an important role in the early development of mouse and human embryos (144, 145). RNA methylation is believed to have a major role in maternal RNA degradation. In Drosophila, METTL3-METTL4 complex-mediated m6A modification contributes to normal embryogenesis and may be involved in the partial degradation of maternal RNA. Additionally, FMR1 preferentially binds RNAs containing the m6A-modified “AGACU” and controls the decay of its target parent mRNA in a m6A-dependent way (146). During the MZT, transcriptionally silenced embryos are dependent on post-transcriptional regulation of maternal mRNA until zygote genome activation (ZGA) (147). There is evidence that the recently identified m6A reader IGF2BP recruits the mRNA stabilizer HuR to prevent the degradation of m6A-containing mRNAs and to promote their translation (44). Maternal mRNA stability in zebrafish has been found to be significantly regulated by IGF2BP3. In zebrafish, cytoplasmic division and cytoskeletal processes are reportedly mediated by AURKB (aurora B). As a target of IGF2BP3, AURKB showed significantly reduced mRNA expression in embryos with IGF2BP3 knockdown, resulting in quick degradation of maternal mRNA (148, 149). The m6A reader YTHDF2 are also proved to primarily affect the methylated maternal mRNA at 4 h.p.f, regulating zebrafish maternal mRNA clearance and thereby controlling development through MZT (149). Further research indicated that the methyltransferase METTL16 is essential for the viability of early mouse embryos by modulating MAT2A mRNA. The loss of METTL16 causes a dramatic change in the E3.5 blastocyst transcriptome, preventing the further development of mouse embryos (150). Most recently, single-cell m6A sequencing (scm6A-seq) methods were used to analyze the m6A methylome and transcriptome in individual oocytes as well as organelles of cleavage-stage embryos. It has been demonstrated that METTL3-catalyzed m6A primarily affects RNA stability and can preferentially promotes degradation in germinal vesicle oocytes. In the field of single-cell research, the findings indicated dynamic m6A regulation in RNA metabolism during oocyte development and syncytial genome activation (151). In addition, the m6A reader YT521-B homology domain protein 1 (YTHDC1) and its target m6A RNAs function upstream of SETDB1 to maintain mouse embryonic stem cells and repress retrotransposons and bicellular phase (152).

After implantation of the embryo in the uterus, RNA post-transcriptional modifications also regulate germ layer growth. The rRNA m6A methyltransferase METTL5 is engaged in the developmental process (153). METTL5 must form a heterodimeric complex with TRMT112 to establish metabolic stability in cells (56). It methylates 18S rRNA both in vivo and in vitro, and METTL5-deposited m6 A is involved in regulating efficient translation of the F-box and WD repeat domain-containing 7 (FBXW7) to enhance mouse embryonic stem cell differentiation (154, 155). In addition, evidence suggests that m6A deposition is particularly necessary for the induction of ectoderm, but not for the differentiation of the stereotyped endoderm. METTL14 promotes ectodermal maturation, which is necessary for post-implantation development in mice. The absence of METTL14 and the associated drop in m6A levels impede the ability of ESC initiation and further differentiation, resulting in the inability of mouse ESC to transition from the naive to the initiating state (156). However, various researchers believe that m6A deposition is equally crucial in the induction of endodermal differentiation. Arginine methylation of METTL14 is a novel molecular mechanism that regulates m6A deposition. m6A deposition is regulated by PRMT1-mediated arginine methylation of METTL14 at its disordered C-terminal region (157). The R255 location of METTL14 was discovered to impact the endodermal differentiation of mouse embryonic stem cells, implying a direct link between protein methylation and RNA methylation (158). Of note, the exact effect of m6A methylation modification of RNA on which germ layer differentiation takes place still needs further study.

Other methylation changes, such as m5C methylation, have been demonstrated to have similar consequences to m6A methylation in early embryonic development.

The majority of m5C sites in protein-coding RNAs are catalyzed by NSUN6. Evidence suggests that embryonic development in mice is largely unaffected by the complete loss of NSUN6. In vitro cellular investigations and in vivo animal assays were carried out for verification. Reduced levels of NSUN6-targeted mRNAs are demonstrated in embryonic-like bodies lacking NSUN6 (120). However, there are currently conflicting perspectives on whether m5c promotes embryonic development. Jianheng Liu emphasized the functional importance of the maternal mRNA m5C by quantitative mRNA m5C maps of six vertebrate and invertebrate species at different developmental stages. They discovered that NSUN2 deletion embryos incur cell cycle stoppage or delay and fail to commence maternal-to-zygotic transition in time using D. mel as a model (159). Moreover, NSUN5 deficiency leads to decreased m5C in the exon and 3’UTR regions as well as a reduction in the translation efficiency of mitotic arrest deficient 2 like 2 (MAD2L2) and growth differentiation factor 9 (GDF9) in the ovary, inhibiting folliculogenesis and development during ovarian aging. This suggested that NSUN5/m5C-regulated maternal mRNA stability is essential for MZT transition as well (160). In zebrafish, Ybx1 and Pabpc1a coordinate to regulate the stability of m5C-modified maternal mRNA. Y-box binding protein 1 (Ybx1) recognizes m5C via π-π interaction with the key residue Trp45 in the Ybx1 cold shock domain (CSD) preferentially C-modified mRNA, which plays a role in maternal mRNA stability and early embryogenesis (161). Additionally, the RNA cytosine methyltransferase NSUN3 is an important factor in ESC differentiation, especially in neuroectodermal differentiation. Inactivation of NSUN3 skews ESC differentiation towards the mesendoderm and affects Wnt signaling and mitochondrial ROS production in embryonic stem cells (162).

Based on m7G methylated tRNA immunoprecipitation sequencing (MeRIP-Seq) and tRNA reduction and cleavage sequencing (TRAC-Seq), increased ribosome occupancy of the corresponding codon in METTL1 knockout mESCs was observed. This suggests an important role for the mammalian m7G tRNA methylome in ESCs (163).

However, the specific effects of RNA methylation on different phases of embryonic development, whether in m5C modification or m7G modification, have yet to be studied. Their different roles and mechanisms in sperm development, egg maturation, MZT, and embryo layer differentiation after embryo implantation remain the focus of future research.

**Figure 3:**
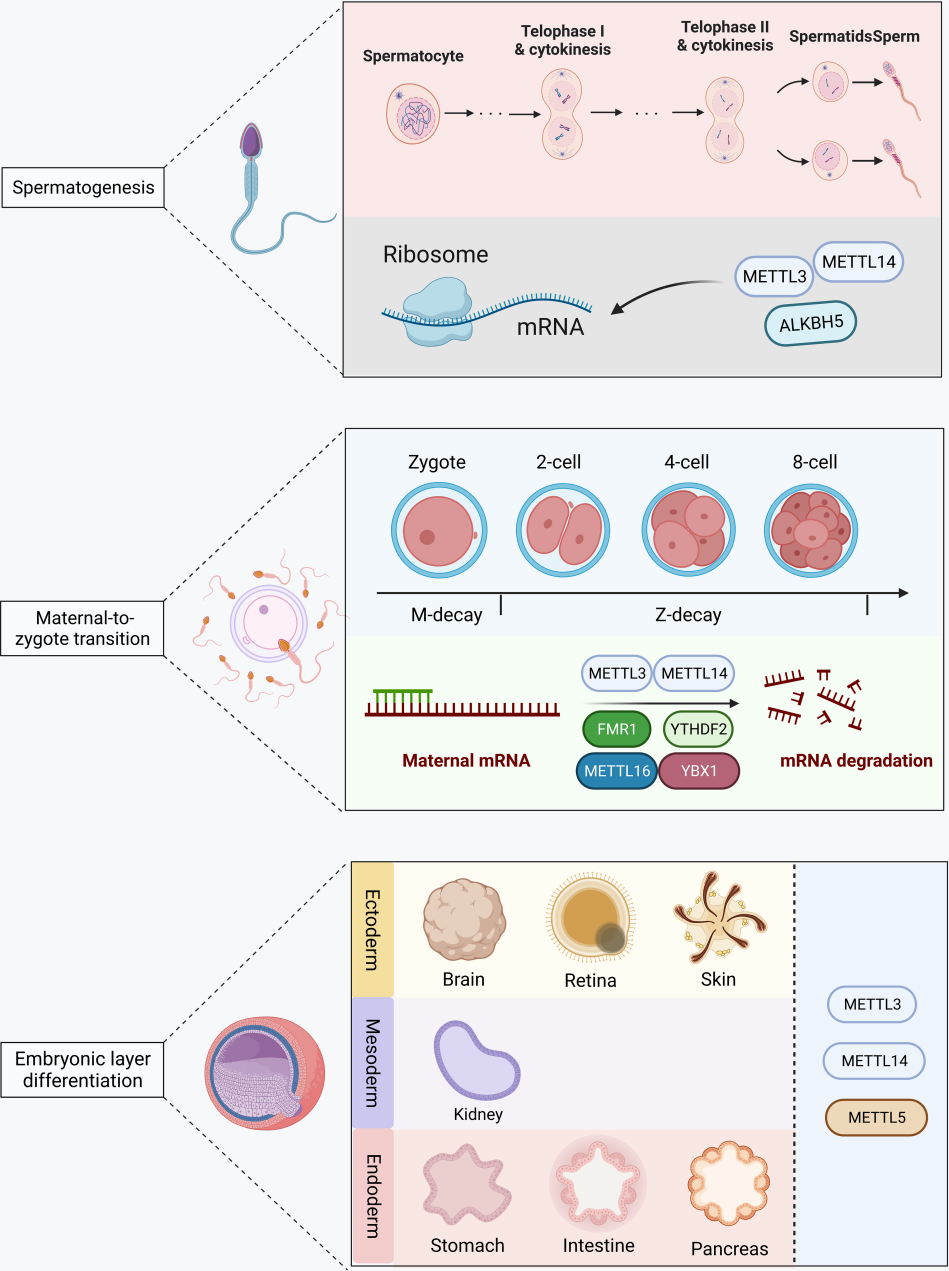
RNA methylation in the early embryo development. METTL3, METTL14, and ALKBH5 modify mRNA and participate in spermatogenesis. METTL3, METTL14, METTL16, FBX1, FMR1, and YTHDF2 are involved in maternal mRNA degradation during the maternal-to-zygote transition. METTL3, METTL14, and METTL15 are involved in germ layer differentiation after embryo implantation.

Overall, RNA methylation is involved in spermatogenesis, egg maturation and MZT, and embryo layer differentiation in early embryo development (**Figure 3**). Further research is still required for the possible different mechanisms of RNA methylation and demethylation in the development of embryos before and after implantation. Moreover, it also needs further exploration of the function of m5C and m7G RNA modification during early embryo development.

### 3.2 RNA methylation in embryo implantation

Embryo implantation is one of the important aspects of maternal-fetal interface formation. The embryo implantation requires the degradation of the extracellular matrix (ECM) and the establishment of uterine receptivity (164). Embryonic trophoblasts are also important for embryo implantation and placental development. The proliferation, differentiation, and invasive activity of trophoblast cells are all linked to successful embryo implantation, placenta formation, and growth and development. During embryo implantation, trophoblast cells adhere to and invade the maternal endometrium and eventually form the placenta. After implantation, trophoblasts invade and remodel the decidual spiral arteries, providing the basis for material exchange and oxygen transport between the fetus and the mother. Angiogenesis occurs at the maternal-fetal interface and plays a key role in fetal development as the embryo grows after implantation. Thus, trophoblast invasion is a hallmark of placental development in early pregnancy and plays an important role in the implantation and formation of the maternal-fetal interface during pregnancy (165). Trophoblast invasion behavior and its regulatory factors have become a hot topic of current research.

Trophoblast invasion can be regulated by dynamic methylation modifications of mRNA. By modulating MYLK expression, METTL3 enhances trophoblast cell invasion (166). Further analysis revealed that RNA methylation may affect trophoblast invasion by influencing mRNA stability. Based on stromal gel invasion assay and transwell migration assays with METTL3 over-expressing and knockdown in HTR-8 cell lines, the reduction of METTL3-mediated m6A modification attenuated the attenuation of ZBTB4 and increased the expression of ZBTB4, thereby impairing the invasive ability of trophoblast cells. m6A modification in RNA is crucial for controlling trophoblast invasion (167). Another m6A methyltransferase, WTAP, promotes trophoblast invasion and metastasis by regulating the stability of HMGN3 mRNA in a m6A-dependent way (168). Reduced m6A modification may result in inadequate early trophoblast implantation and produce poor pregnancy outcomes. By comparing ALKBH5 and m6A mRNA methylation levels in patients with recurrent miscarriage and normal population, Xiao-Cui Li et al. found that ALKBH5 controls CYR61 mRNA stability by regulating m6A methylation and regulates early gestational trophoblast invasion through its effect on mRNA m6A methylation. CYR61 mRNA levels in villi were inversely linked with ALKBH5 mRNA levels, and ALKBH5 knockdown significantly promoted cell invasion (169). DNA microarray analysis of endometrial cells revealed that IGP2BP family molecules (m6A readers), such as IGF2BP1 and IGF2BP3, were upregulated in human trophoblast ectoderm cells and receptive endometrium cells during the implantation window (170). The relationship between embryo implantation and the IGF2BP growth factor properties or the methylation effect, however, has not yet been thoroughly investigated. In addition to m6A methylation, other modifications in RNA also work in the biological behaviors of trophoblast cells. 5-hmC RNA methylation is also associated with cell invasion and early placental formation. In early pregnancy, hypoxia is the typical extrinsic factor regulating trophoblast migration invasion and placental formation. TET1 is an enzyme that converts 5-mC to 5-hydroxymethylcytosine (5-hmC). By simulating the physiological hypoxic conditions of early pregnancy, TET1 was found to be activated in cells exposed to 3% O2, and TET1 knockdown inhibited cell migration and invasion (171). It has also been proposed that m1A methylation influences trophoblast invasion. YTHDF3 selectively interacts to m1A-containing RNA, and hypoxia downregulates its expression in trophoblast HTR8/SVneo. YTHDF3 suppresses IGF1R expression by binding to m1A modifications and enhances IGF1R mRNA attenuation, inhibiting MMP9 expression and, as a result, trophoblast invasion (172).

These studies demonstrated that trophoblast cell invasion is mediated by revisable RNA methylation. Additionally, RNA methylation alterations encourage trophoblast invasion, while demethylation modifications prevent it. Nonetheless, a few considerations challenge the hypothesis. Evidence shows that hypoxia treatment can significantly upregulate ALKBH5 expression and induced ALKBH5 translocation from the nucleus to the cytoplasm, followed by ALKBH5 demethylation of m6A modification on SMAD1/5 mRNA, which promoted its expression and activated the TGF-β signaling pathway to induce MMP9 expression, thereby increasing trophoblast migration and invasion (173). ALKBH5 has also been demonstrated to reduce the m6 A level of PPARG mRNA and increase the stability of PPARG mRNA, hence increasing PPARG translation. PPARG overexpression promotes trophoblast cell proliferation and migration through the upregulation of ALCAM expression (174). Another study revealed that overexpression of METTL14 in HTR8/SVneo cells inhibited trophoblast proliferation and invasion by increasing FOXO3a expression (175). These results reveal the complexity of the regulation of trophoblast invasion by RNA methylation.

Non-coding RNA methylation also affects trophoblast cell proliferation and invasion. YTHDC1, for example, degrades circMPP1 via mA6 alteration, enhancing placental villi function (176). The m6A modification of circSETD2 represses miR-181a-5p and increases MCL1 transcription, thereby regulating trophoblast cells (177).

Mounting evidence implies that B(a)P/benzo(a) pyrene-7,8-dihydro diol-9,10-epoxide (BPDE) inhibits the proliferation of human chorionic trophoblast cells and is related to RNA m6A modification. Mengyuan Dai e al. discovered a novel lncRNA, lnc-HZ09, and its putative function in recurrent miscarriage villous tissue and human trophoblast cells exposed to BPDE. Cells over-expressing METTL3 have higher levels of m6A RNA modification on lnc-HZ09 and result in reduced stability of lnc-HZ09. lnc-HZ09 regulates trophoblast migration and invasion through the PLD1/RAC1/CDC42 pathway, and lnc-HZ09-silenced cells exhibit significantly greater migration and invasion (178). In addition, lnc-HZ01 and METTL14 were also shown to be upregulated in the recurrent miscarriage group compared to the normal group in villi tissue. METTL14 levels were positively correlated with m6A methylation levels on lnc-HZ01 levels. The downstream MXD1/EIF4E pathway is activated, which reduces trophoblast growth and causes miscarriage (179).

These findings highlight the significance of RNA methylation in controlling the biological behaviors of trophoblast cells. However, whether RNA methylation changes on trophoblast cells promote or impede invasion is debatable. Furthermore, the majority of the aforementioned pathways were discovered during the aberrant trophoblast implantation experiments. It is unclear whether these mechanisms have a role in normal trophoblast invasion and maternal-fetal interface establishment during early pregnancy.

### 3.3 RNA methylation in decidualization

Decidualization refers to the functional and morphological changes occurring in the endometrium. The main changes that occur during metaplasia are changes in the endometrial stromal cells, increased uterine gland secretion, increased uterine natural killer cells, and vascular remodeling. Accumulating experimental and clinical evidence demonstrated that defective decidualization or blocked decidualization may lead to abnormal maternal-fetal interface (180).

Endometrial stromal cells are the most common type of cell involved in decidualization. During embryo implantation, METTL3 is necessary for the decidualization of human endometrial stromal cells in vitro. Progesterone receptor (PGR) mRNA is a direct target of METTL3-mediated m6A modifications. In METTL3^f/f^ mice, the m6A-modified PGR mRNA in 5 ‘- UTR is recognized by YTHDF1, thereby promoting PGR protein translation. However, in Mettl3^d/d^ mice, the m6A modification in PGR mRNA is missing, and the PGR protein cannot be effectively translated. The low level of PGR protein ultimately leads to implantation and decidualization failure. The METTL3-PGR axis may be conserved between mice and humans (181). Of note, overexpression of METTL3 increases m6A methylation and destroys embryo attachment by inhibiting the expression of endometrial receptive biomarker HOXA10 (182).

Additionally, a major hallmark of decidualization is the mesenchymal-epithelial transformation (MET) of endometrial mesenchymal cells (183). The transformation between mesenchymal and epithelial was strongly correlated with RNA methylation. METTL3 enhances ZMYM1 mRNA expression through an m6A-HuR-dependent pathway, and epigenetic activation of ZMYM1 can lead to epithelial-mesenchymal transformation (EMT) (45). m6A-seq data revealed that during EMT, m6A modification occurs on a few genes related to cell attachment, adhesion, and migration (184). However, the effect of RNA methylation on the transition between mesenchyme and epithelium has not been demonstrated in the endometrium. Whether RNA methylation promotes decidualization through MET remains a mystery.

Collectively, the above findings imply that m6A methylation is linked to the decidualization of human endometrial stromal cells and may potentially be linked to MET. More research, however, was required to confirm the rationale behind these findings.

### 3.4 RNA methylation of the immune microenvironment at the maternal-fetal interface

The immune microenvironment at the maternal-fetal interface is essential for maintaining normal embryonic development. Decidual natural killer (dNK) cells are the most prevalent in the maternal-fetal interface, followed by decidual macrophages (dM Φ) and then decidual T cells (185).

There is evidence that m6A methylation and NK cell effector function are linked to METTL3 expression levels. METTL3-mediated m6A methylation protects the signaling pathway downstream of IL-15R, thereby regulating the responsiveness of NK cells to IL-15 (186). NPM1 is associated with m6A methylation and immune infiltration. Its expression is linked to B-cell and NK-cell marker genes (187). However, there is currently no direct evidence to prove the influence of RNA m6A modification on dNKs.

RNA methylation can affect macrophage polarization. The m1A “reader” YTHDF3 may be involved in regulating macrophage polarization that promotes aortic inflammation (188). According to the findings of the ssGSEA analysis, the maternal-fetal interface comprises a substantial number of immune-related cells, with ALKBH5 serving as the key m6A regulator of macrophages. Overexpression of ALKBH5 in ESC affects macrophage differentiation through VEGF secretion (189). Mechanically, METTL3 can drive M1 macrophage polarization through direct methylation of STAT1 mRNA and may serve as an anti-inflammatory target (190).

RNA methylation also affects TCR signaling and determines T cell activation and survival. WTAP and m6A methyltransferase functions are required for thymocyte differentiation and control of activation-induced peripheral T-cell death (191). By targeting the IL-7/STAT5/SOCS pathways, m6A mRNA methylation controls T cell homeostasis (192). It also maintains Treg inhibition through the IL2-STAT5 signaling axis (193). In addition, TRMT6/TRMT61A complex-mediated m1A modification of transfer RNA (tRNA) promotes the synthesis of MYC proteins essential for T cell proliferation (194, 195). This demonstrates that m1A methylation also has important effects on T cells.

Based on the above findings, we propose that RNA methylation may have important effects on the immune microenvironment. Changes in NK cell function, macrophage polarization, and T cell activation are all linked to RNA methylation. Although the majority of the current studies on the impact of RNA methylation in the immune microenvironment focus on the tumor immune microenvironment, these studies can provide some insight into the role of RNA methylation in the maternal-fetal immune microenvironment to a certain extent.

Taken together, due to its ability to promote embryo development, regulate embryo implantation, extend uterine receptivity, and provide a stable immune microenvironment, RNA methylation is important in the formation of the maternal-fetal interface.

## 4 RNA methylation in implantation and placentation-related diseases

Common implantation and placentation-related diseases like miscarriage, preeclampsia, and preterm birth have been proposed to be associated with aberrant RNA methylation in the formation of the maternal-fetal interface. Abnormalities in RNA methylation may impact the establishment of the maternal-fetal contact, ultimately leading to illness.

### 4.1 Miscarriage

Miscarriage, especially recurrent miscarriage, may be multifactorial. Problems in any part of the maternal-fetal interface formation can lead to miscarriage. In this process, RNA methylation probably plays an important role. It was discovered that m6A levels in the uterus rise throughout pregnancy. The mRNA levels of the methyltransferases METTL16 and WTAP were lower in the endometrium of patients with miscarriage and infertility, whereas the mRNA levels of ALKBH5 and IGF2BP2 were higher (196).

In the process of early embryonic development, abnormal methylation in epigenetic factors of both the father and mother can affect embryonic development (197). Abnormal RNA methylation plays a role in abnormal embryo implantation and may also lead to spontaneous abortion. High-throughput sequencing of villous tissue from the spontaneous abortion group and the normal group during early pregnancy, based on combined analysis of meRIP-seq and RNA-seq data, revealed that genes with differential m6A methylation were primarily related to the Wnt signaling pathway, phosphatase activity regulation, protein phosphatase inhibition activity, and transcriptional inhibition activity (198). The expression levels of ALKBH5 and CYR61 were negatively correlated in the villous tissue of the recurrent abortion group, which is related to the pathogenesis of recurrent abortion (169).

Moreover, FTO deficiency in the chorionic villi affects immunological tolerance, leading to aberrant methylation and oxidative stress and, eventually, spontaneous abortion. Compared with normal pregnant women, the levels of FTO-binding HLA-G, VEGFR, and MMP9 mRNA in spontaneous abortion patients were significantly reduced, and the concentration of FTO-binding MMP7 was significantly increased. This is due to oxidative stress and abnormal m6A accumulation at the maternal-fetal interface (199).

These all prove that abnormal methylation of RNA might be one of the reasons for spontaneous abortion.

### 4.2 Preeclampsia

Preeclampsia is classified as early-onset or late-onset based on when it appears, which is usually at 34 weeks of gestation. Early-onset preeclampsia often results in a high perinatal mortality rate, significant placental dysfunction, and poor maternal and fetal prognosis. Our review focuses on early-onset preeclampsia because it is the most severe and is a major cause of maternal and fetal death. It is strongly associated with RNA methylation. Trophoblast dysfunction is thought to be an important cause of early-onset preeclampsia, and RNA methylation has a major impact on trophoblast function in two ways, according to the existing studies.

On the one hand, RNA methylation influences trophoblast invasion in patients with preeclampsia. We discovered that DNMT3B and TET3 were significantly elevated in PE samples compared to the normal group by analyzing the mRNA expression levels of m5C RNA methylation regulators. Surprisingly, these two methylation regulators have a negative correlation. A comprehensive analysis of UMI-MeRIP Seq data revealed a decrease in the overall peak value of m5C methylation in placental tissue from patients with preeclampsia. However, compared with the normal blood pressure group, the m5C peak value before the coding sequence was stopped in the preeclampsia group significantly increased (200). In addition, preeclampsia placental samples exhibited consistently increased SMPD1 protein levels and increased 5’-UTR of m6A (201). Evidence suggests that m6A RNA methylation expression is significantly increased in trophoblast cells of preeclamptic placentas compared with normotensive placentas and that abnormal m6A modification may contribute to trophoblast cell invasion dysfunction in preeclampsia (202). An increase in m6A-modified circRNA was found to be prevalent in the PE placenta. The m6A-modified circPAPPA2 has a role in trophoblast invasion (203). Several evidences have shown that the downregulation of m6A reader IGF2BP2 is associated with trophoblast invasion disorder and PE. For example, there is a correlation between reduced IGF2BP2 expression and downregulation of MNSF in the placenta of MNSF-deficient mice and patients with severe (204). However, it is yet unknown whether methylation has a role in how IGF2BP affects PE.

RNA methylation, on the other hand, induces trophoblast apoptosis. YTHDF2 can target TMBIM6 mRNA via METTL3, resulting in ROS production and trophoblast apoptosis, exacerbating endoplasmic reticulum stress in preeclampsia (205). Similarly, m6A modification promotes circSETD2 expression, suppresses miR-181a-5p, and increases MCL1 transcription to affect the apoptosis of chorionic trophoblast cells (177).

In short, in patients with preeclampsia, a decrease in the overall peak of m5C methylation or an increase in m6A modification may lead to trophoblast invasion dysfunction. In addition, increased RNA methylation can promote trophoblast cell apoptosis, further exacerbating preeclampsia. Interestingly, the methylation trends of m5C and m6A in preeclampsia appear to be different. In addition, in the process of m5c methylation regulation, DNMT3B and TET3 seem to have opposite effects, but they are highly expressed in preeclampsia patients. In this process, the molecular mechanism of regulators is still unknown.

### 4.3 Preterm birth

Preterm birth is defined by the World Health Organization (WHO) as delivery before 37 completed weeks of gestation. Between 25% and 40% of all preterm births are associated with intrauterine infection, including viral and bacterial infections (206).

m6A factors can regulate the virus life cycle. By adding m6A to the genome RNA and mRNA of a virus, changing gene expression, or the fate of corresponding RNA involved in virus or replication, the effect of m6A modification can directly or indirectly regulate virus replication. The strength and duration of innate immune response activation can also be determined by m6A methylation (207). m6A-modified HBV RNA can be coupled to YTHDF2, which can protect viral RNA from being recognized by RIG-I and prevent HBV RNA degradation (208). During the infection cycle, HSV-1 controls the redistribution of the nuclear m6A mechanism in primary fibroblasts. WTAP stays in the nucleus, while METTL3 and METTL14 are distributed throughout the cytoplasm (209). Based on the role of m6A modification in viral infection, m6A modification can be used to develop anti-viral methods. For instance, interferon-stimulating gene 20 (ISG20) can treat HBV infection by selectively recognizing and processing m6A-modified HBV transcripts, and regulated by YTHDF2 (210).

Bacterial infections, in addition to viral infections, can result in premature delivery. The P/Q/N rich domain in m6A reader YTHDF1 interacts with the DEAD domain in host factor DDX60, thereby regulating the immune response to bacterial infection by recognizing the target Traf6 transcript (211).

Moreover, in patients with spontaneous preterm delivery, abnormalities in immune function at the maternal-fetal interface may be a major cause of their preterm delivery (212). The association between RNA methylation and the immune microenvironment has primarily been examined in malignancies, but whether there is a similar relationship at the maternal-fetal interface still needs to be investigated.

Currently, research in the field of RNA modification in preterm birth is mainly focused on infection. The m6A methylation is the most common type of RNA alteration involved. However, whether or not RNA methylation alteration is one of the major biochemical pathways causing premature delivery is still being contested.

Above all, an imbalance in RNA methylation is critical in the development of implantation and placental disorders. Interestingly, the newly discovered programmed cell death pathways, including autophagy, ferroptosis, and pyroptosis are also associated with implantation and placenta disorders. Recently, autophagy has shown to have a role in miscarriage. In women who have had an early abortion, dysregulated autophagy promotes oxidative stress and aberrant production of ABC transporter proteins (213). Many individuals with unexplained spontaneous abortion have insufficient autophagy (214). Rapamycin, a known inducer of autophagy, was found to dramatically boost endometrial autophagy and NK cell residency, as well as improve spontaneous abortion in mice (215). Ferroptosis and pyroptosis have also been linked to preeclampsia in recent studies. Preeclampsia is characterized by hypoxia, which causes iron death of trophoblast cells via apoptosis, autophagy, and necrosis. Iron death-related proteins SRXN1 and NQO1 may be important in the etiology of preeclampsia (216). In early-onset pre-eclamptic placentas and human trophoblast cells exposed to hypoxia and endoplasmic reticulum stresses, pyroptosis is suggested to represent a significant inflammatory mechanism (217). Current evidence suggests that impaired autophagy and increased apoptosis in a placental microenvironment with Th1/Th2 imbalance are associated with spontaneous preterm birth (218).

There is currently a growing body of evidence linking RNA methylation to programmed cell death. m6A methylation is associated with autophagy and plays a role in apoptosis induced diseases. Silencing METTL3 enhances autophagic flux and inhibits apoptosis in H/R-treated cardiomyocytes (219). Moreover, the role of m6A modification and autophagy is of great importance in inflammatory diseases and cancer progression. METTL3-mediated m6A modification of ATG7 regulates the autophagy-GATA4 axis and promotes cellular senescence and osteoarthritis (220).

However, it is unclear whether RNA methylation affects implantation and placenta-associated diseases by influencing programmed cell death. As a result, more evidence is needed to demonstrate whether RNA methylation-mediated programmed death is directly involved in these diseases.

## 5 Conclusions and future directions

This is the first description of the dynamic RNA methylation function during the maternal-fetal interface development. We performed in-depth investigations of RNA methylation for this review. The three most frequent RNA modifications, m6A, m1A, and m5C, are all essential for the biogenesis, metabolism, structural stability, and function of RNA.

RNA methylation may have a considerable impact on the creation of the maternal-fetal interface by influencing early embryonic development, embryo implantation, endometrial receptivity, and the immunological microenvironment. Furthermore, abnormal RNA methylation during the formation of the maternal-fetal interface is thought to be associated to implantation and placenta-related disorders such as miscarriage, preeclampsia, and preterm birth.

Given the significance of RNA methylation in the formation of the maternal-fetal interface during pregnancy, further investigation is warranted to elucidate the underlying mechanism. The potential various mechanisms of RNA methylation and demethylation during the development of embryos before and after implantation still require further research. Furthermore, there are differing views on whether trophoblast cell RNA methylation changes encourage or impede invasion. The majority of RNA methylation mechanisms in trophoblast cells have been discovered in studies of abnormal trophoblast implantation, but it is still unknown whether these mechanisms contribute to normal trophoblast invasion and the establishment of the maternal-fetal interface in the early stages of pregnancy. There is also no direct evidence for the effects of RNA methylation on the immunological milieu at the maternal-fetal interface. It is noteworthy that whereas m6A methylation has been linked to EMT and metaphase, the underlying regulatory mechanism is yet unknown.

Additionally, it is unclear whether RNA methylation affects implantation and placenta-associated diseases by influencing programmed cell death. From a broader perspective, the role of RNA methylation and programmed cell death in implantation- and placentation-related illnesses is another significant area of challenge and promise for future research.

Together, our study highlights the importance of dynamic RNA methylation at the maternal-fetal interface. It has given the molecular underpinnings for both normal and abnormal implantation and placentation and could eventually offer a fresh approach to treating related disorders.

## Author contributions

HY and YJ designed the work and made major revisions to the article. SW and HX wrote the manuscript. YS, XJ, and YM drafted the manuscript and revised the article. All authors contributed to the article and approved the submitted version.
